# The Human Breast Milk Metabolome in Overweight and Obese Mothers

**DOI:** 10.3389/fimmu.2020.01533

**Published:** 2020-07-21

**Authors:** Flaminia Bardanzellu, Melania Puddu, Diego Giampietro Peroni, Vassilios Fanos

**Affiliations:** ^1^Neonatal Intensive Care Unit, Department of Surgical Sciences, AOU and University of Cagliari, Monserrato, Italy; ^2^Clinical and Experimental Medicine Department, Section of Pediatrics, University of Pisa, Pisa, Italy

**Keywords:** breast milk, breastfeeding, obesity, overweight, gestational diabetes mellitus, diabetes, metabolomics

## Abstract

Pre-pregnancy body mass index (BMI) is a major relevance factor, since maternal overweight and obesity can impair the pregnancy outcome and represent risk factors for several neonatal, childhood, and adult conditions, including excessive weight gain, cardiovascular disease, diabetes mellitus, and even behavioral disorders. Currently, breast milk (BM) composition in such category of mothers was not completely defined. In this field, metabolomics represents the ideal technology, able to detect the whole profile of low molecular weight molecules in BM. Limited information is available on human BM metabolites differences in overweight or obese compared to lean mothers. Analyzing all the metabolomics studies published on Medline in English language, this review evaluated the effects that 8 specific types of metabolites found altered by maternal overweight and obesity (nucleotide derivatives, 5-methylthioadenosine, sugar-alcohols, acylcarnitine and amino acids, polyamines, mono-and oligosaccharides, lipids) can exert on the risk of offspring obesity development and other potentially associated health outcomes and complications. However, metabolites variations in samples collected from overweight and obese mothers and the potentially correlated effects highlighted below still need further investigations and should be confirmed in future metabolomics studies on larger samples. Finally, the positive or negative influence of maternal overweight and obesity on the offspring, potentially exerted by breastfeeding, should be analyzed in close correlation with maternal age, genetic and environmental factors, including diet, and taking into account the interactions occurring between BM metabolites and lactobiome. The evaluation of all the factors affecting BM metabolites in overweight and obese mothers can lead to the comprehensive description of such biofluid and the related effects on breastfed subjects, potentially highlighting personalized needs of BM supplementation or short- and long-term prevention strategies to optimize offspring health.

## Introduction

Obesity is a growing social problem affecting an increasing number of women in reproductive age. It represents a risk factor potentially impairing the pregnancy itself, and even the long-term outcome of the offspring, since it is well known that the overweight condition can be transmitted to the future generations. Several observational studies and some meta-analyses have been carried out to assess whether breastfeeding is positively correlated with a reduction in the incidence of obesity in later life. Although most of them showed a modest reduction in such risk (1), rigorous reviews (1–3) conclude that there is no clear cause-effect relationship, because of several confusing factors and frequent bias of such studies. Currently, conditions as the socio-economic status of the mother, the level of education, ethnicity, eating habits of the family, and duration of breastfeeding have not been considered and could themselves justify the association. On the other hand, a study reports that the risk of obesity is higher in the non-breastfed children of obese mothers, but remains higher even in those who are breastfed than in the general population (4).

Nutrition in the early stages of life plays a fundamental role in the child's growth and development, and is presumably one of the main players in the “programming” of his future health. In the very early phases of life breast milk (BM) can affect the maturation of organs and systems, influencing the future health.

Human BM is a complex biofluid containing a very large number of components, including macronutrients, hormones, bioactive molecules, stem cells, and microbial communities; each of them is potentially responsible for a certain specific and even synergic influence on the newborn outcome, on its growth and on the development of organs and systems, as deeply reviewed in several paper by ours (5–10).

It could be hypothesized that maternal pathological conditions could influence BM composition, but there are few studies in agreement with these considerations. In a recently published review by our group, to our knowledge the first on this topic, the studies using metabolomics to investigate BM of women with great obstetrical syndromes were discussed; it emerged that some metabolites seem to differentiate BM of healthy women and samples collected from women with preeclampsia, gestational diabetes, and intrauterine growth restriction; these metabolites might be related to the long-term outcomes in the offspring of affected mothers. The results of the analysis seem to highlight the involvement of the mammary gland in the underlying pathological processes and suggest the possibility that BM, while remaining the food of first choice in the early stages of life, could benefit from targeted supplementation to promote a better infant outcome (11).

Up to now, a clear description of BM composition in overweight or obese mothers has not been provided. Metabolomics is an emerging method in the study of BM and has proved useful in differentiating the characteristics of healthy women milk according to gestational age and lactation stage (12–14).

In the present paper, through the review of the metabolomics studies on BM collected from overweight-obese women, for the first time, we aim to investigate the metabolites found altered in the different studies, to assess whether they can have a positive (or negative) effect on the onset of obesity and other long-term complications.

We accurately searched on Medline the whole available literature, in English language, applying metabolomics to characterize BM in overweight and obese mothers; thus, breast milk, overweight, obesity, metabolomics, lipidomics, and oligosaccharides were used as key words.

As result, we found and discussed a single article on untargeted metabolomics (15), a total of four articles on targeted metabolomics (16–19), and 10 articles on lipidomics (20–29) published since 2013–2020. Review articles were excluded.

The main metabolomics differences detected by comparing BM of overweight/obese mothers and lean ones, and the potential short- and long-term effects in offspring have been summarized in Table 1. When available, details on maternal age in the different studies have been reported in the footnotes.

**Table 1:** Main metabolomics differences detected by comparing breast milk of overweight/obese mothers and lean ones in the different studies, and the potential short- and long-term effects in offspring.

**Metabolites variation in overweight/obese mothers samples**	**Potential long term effects in offspring**
**PYRIMIDINE DERIVATIVES**	
**Orotate** ↓ at 1 month of lactation (15)	Altered glucose homeostasis More weight gain by an inadequate diet Negative effect on the development of immune processes
**PURINE DERIVATIVES**	
**AMP, Adenine** ↑ at 1 month of lactation (15)	↑ Overweight risk Protection from obesity associates insulin-resistance Positive effect on the development of immune processes ↑ Neuroprotection ↓ Cardio-vascular risk
**Methylthioadenosine** ↑ at 1 month of lactation (15)	Protection against cardio-metabolic risk
**SUGAR ALCOHOLS**	
**Erythritol** ↑ at 1 month of lactation (15)	↑ Overweight risk
**AMINOACIDS (AND ACYLCARNITINES)**	
**Branched chain aminoacids (BCAAs)** ↑ at 3 month of lactation (16)	↑ Cardio-metabolic risk Unfavorable neurological outcomes
**3-5Acylcarnitines (ACs)** ↑ at 6 month of lactation (15)	
**Glutamine** ↓ at 6 months of lactation (15)	Altered glucose homeostasis Unfavorable neurological outcomes (as precursor of glutamate)
**Asparagine** and **Ornithine** ↓ at 6 months of lactation (15)	↑ Cardio-metabolic risk
**Aromatic aminoacids and derivatives**	
**Tyrosine** ↑ at 6 months of lactation (16)	↑ Cardio-metabolic risk
**Kynurenic acid** ↓ at 6 months of lactation (15)	Protection against cardio-metabolic risk from oxidative stress and inflammation
**2-Aminobutyrate (2-AB)** ↑ at 1 month of lactation (15)	Protection against oxidative stress
**Polyamines** ↓ at 3 days, 1 month and 6 months of lactation (17)	Less protection against cardio-metabolic risk from oxidative stress and inflammation Less neuroprotection
**MONOSACCHARIDES**	
**1-5 anhydroglucitol (1,5-AG)** ↑ at 1 and 6 months of lactation (15)	Emerging hyperglycemia marker **In breast milk** Potential role in describing maternal glycemic control
**Arabinose** ↑ at 6 months of lactation (15)	Effects on some pathogens, potentially reducing their virulence
**Glucose-6-phosphate** ↑ at 6 months of lactation (15)	Protection against oxidative stress Providing of energy supply
**OLIGOSACCHARIDES**	
**Lacto-N-fucopentaose I** ↓ at 1 month of lactation (15)	↑ Overweight risk ↓ Infant height ↓ Protection against infections Negative influence on neonatal gut microbiota, i.e., reducing *Lactobacillus* spp. (30)
**Lacto-N-fucopentaose II** ↑ at 1 month of lactation (15)	↑ Overweight risk
**Lacto-N-fucopentaose III** ↑ at 1 month of lactation (15)	↑ Infant height promotion ↑ Protection against infections ↑ Gut content of *Lactobacillus* spp. (30)
**2′-Fucosyllactose** ↓ at 1 month of lactation (15) Higher in *Se+* overweight mothers than *Se+* non-overweight ones (observation not confirmed in obese mothers) (18)	No clear associations with infant growth Its reduction could lead to: ↓ Infant weight, height and growth promotion ↓ Protection against infections
**3′-Fucosyllactose** ↑ at 1 month of lactation (15) Lower in *Se+* overweight mothers than *Se+* non-overweight ones. (observation not confirmed in obese mothers) (18)	No clear associations with infant growth
**Lacto-N-hexaose** ↓ at 3-4 months of lactation (19)	↓ Overweight risk
**LIPIDS**	
**Saturated fatty acids** ↑ At 1 and 2 months (23, 24), and at 3 months of lactation (21)	↑ Weight and BMI gain up to 13 months
**Palmitic acid (16:0)** ↑ at 2 weeks of lactation (25) **Tridecanoic acid (C13:0)** ↑ in colostrum (26)	↑ Overweight risk ↓ Glucose tolerance ↓ Insulin response ↓ Oxidation of fatty acids ↑ Inflammatory and metabolic responses
**MUFA/SFA, UFA/SFA** ↓ at 3 months of lactation (21)	↑ Weight and BMI gain up to 13 months
**Total MUFA** ↓ at 1–3 months of lactation (22, 23) **Oleic acid (18:1)** ↓ at 2 weeks of lactation (25)	↑ Overweight risk Worsening of metabolic and lipid profiles
**n3 PUFA** ↓ at 1–3 months (21, 22, 29) and at 6–7 months of lactation (20) ↓ from 3 days to 2 months of lactation (24) ↑ in colostrum (26)	↑ Overweight risk ↑ Inflammation
**ALA, EPA, DHA** ↓ at 1–3 months of lactation (22, 23, 29) and from 3 days to 2 months of lactation (24)	↑ Overweight risk Unfavorable sensorineural outcome
**n-6 PUFA** ↑ at 2 months of lactation (29) and at 6–7 months of lactation (20) **DGLA** ↑ at 2 weeks of lactation (25)	↑ Weight for age z-score ↓ Lengh for age z-score and CC between 2 weeks and 2 months of age ↑ Inflammation
**n-6/n-3** ↑ at 1–3 months of lactation (21–23, 29) and from 3 days to 2 months of lactation (24)	↑ Overweight risk ↑ Inflammation
**Adrenic acid (22:4 n-6)** ↑ in colostrum (26) and at 2 weeks of lactation (25)	Promotion of CNS development
**PAHSA levels** ↓ at 3 days of lactation (28)	↑ Overweight risk ↑ Inflammation ↓ Glucose tolerance
**Conjugated linoleic acid isomers** ↑ at 2 weeks of lactation (25) ↓ in colostrum (26)	Conflicting results in the 2 available studies (25, 26)

*UFA, unsatured fatty acids; SFA, satured fatty acids; MUFA, mono-unsaturated fatty acids; PUFA, unsaturated fatty acids; ALA, α-linolenic acid; EPA, eicosapentaenoic acic; DHA, docosahexaenoic acid; DGLA, dihomo-gamma-linolenicacid; PAHSA, palmitic acid ester of hydroxystearic acid*.

*When available, details on maternal age in the different studies have been reported in the footnotes*.

**Details on maternal age (means±SDs):**

(15): Average age of overweight-obese mothers: 30.5 ± 4.7 years (no statistically significant differences with lean group).

(16): Average age of obese mothers: 30.2 ± 4.7 years (no statistically significant differences with lean group).

(17): Average age of obese mothers: 30.2 ± 5.8 years (no statistically significant differences with lean group).

(18): Average maternal age is reported according to Secretor status, not referring to maternal BMI.

(19): Average age of the mothers in the study: 33.0 ± 4.2 years (considering overweight and lean mothers together).

(20): Average age of overweight mothers: 34.06 ± 3.37 years (no statistically significant differences with lean group).

(21): Average age of overweight mothers: 31.0 ± 5.0 years (no statistically significant differences with lean group).

(22): Maternal age in relation to BMI is not reported.

(23): Average age of overweight-obese mothers: 32.0 ± 4 years (no statistically significant differences with lean group).

(24): Average age of lean mothers: 32.0 ± 4.1 years; Average age of obese mothers: 30.5 ± 5.7 years.

(25): Average age of overweight-obese mothers: 29.9 ± 3.8 years (no statistically significant differences with lean group).

(26): Maternal age in relation to BMI is not reported.

(28): Average age of obese mothers: 35.1 ± 4.3 years (statistically higher than lean group).

*(29): Average age of obese mothers: 30.0 ± 5.7 years (no statistically significant differences with lean group)*.

## Nucleotide Derivatives

The effects of nucleotides as bioactive substances in the regulation of body functions have been known since long time. *In vitro* and *in vivo* studies showed that nucleotides can promote gut maturation, affect immune modulation enhancing infant antibody response and, in neonatal gut, they can favor the growth of bifidobacteria. In humans, they are considered semi-essential dietary elements, due to the poor capacity of some tissues to synthesize them *de novo*, such as intestinal mucosa and hematopoietic cells. Even if their addition is optional, their pre-constitute mixes are usually present in infant formula milk, to optimize products resembling more accurately mother's samples (31).

*Pyrimidine derivatives*: in the study by Isganaitis et al., the only one, to our knowledge which performs an untargeted metabolomics analysis, liquid chromatography-gas chromatography-mass spectrometry (UHPLC-GC-MS) was applied to BM analysis at 1 and 6 months post-partum; samples collected from women with BMI > 25 Kg/m^2^ were compared with a control group of lean mothers. Pathway analysis indicated that metabolites related to purine and pyrimidine metabolism were the most represented among those found to be significantly different at 1 month post-partum in BM of overweight-obese mothers compared to normal weight controls (15).

Among pyrimidine derivatives, orotate was reduced in the milk of obese-overweight mothers by about 25% (15). Orotate, introduced with food (especially dairy products) or synthesized *de novo* (from glutamine, ATP and CO_2_), is an intermediate metabolite of pyrimidine synthesis and a precursor of uridine-mono phosphate (UMP), a nucleotide that plays a central role in different aspects of human metabolism (32). BM contains less orotate compared to milk of other species, and mammary gland is assumed to produce it and to have a high rate of UMP synthetase, an enzyme involved in the transformation of orotate into UMP, readily absorbed in gastro-intestinal tract (33). UMP, in addition to the involvement in nucleic acids synthesis, is the precursor of uridine-di-phosphate (UDP)-sugars, extracellular signaling molecules whose role in inflammatory and immune processes and in obesity-related glucose metabolism has been recently partially clarified.

With regard to the former, UDP-sugars are the major agonist of P2Y14 receptor (P2Y14R), abundantly expressed in leukocytes and other immune/inflammatory cells. They are also involved in the maturation of dendritic cells, in the degranulation of mast cells, and in the promotion of the regenerative processes of hematopoietic cells in the bone marrow (34). These observations suggest that the reduction of orotate in BM of overweight-obese mothers could have an adverse effect on the neonatal immune processes.

With specific reference to obesity, studies in mice models highlighted the abundant presence of P2Y14R mRNA in the pancreas and its implication in the modulation of insulin secretion. Moreover, the agonist action of UDP-sugars on the P2Y14R would seem to blunt the effect of an high fat diet on weight gain (35). In agreement with these reports, the reduction of orotate in BM of obese-overweight mothers could have a long-lasting adverse effect on glucose tolerance and on the protection, exerted by these nucleotide derivatives, against weight gain promoted by an inadequate diet.

*Purine derivatives*: among the metabolites belonging to purine pathway, adenosine mono phosphate (AMP) and its catabolite adenine were increased in BM of overweight-obese mothers 1 month after birth, while adenosine monophosphate cyclic (cAMP) was reduced (15).

Once introduced with the diet, purine nucleotides no longer necessary for cellular functions are degraded by intestinal enzymes to uric acid. In BM, they can derive from the direct passage from blood to milk or from metabolic processes in the mammary gland (31).

Purines are involved in physiological and pathological processes in all tissues and specifically in adipose tissue. In particular, ATP and adenosine binds to specific receptors of white and brown adipose tissue, both in adipocytes and stromal cells, and their function may be altered in various diseases such as metabolic syndrome (36, 37).

Some of their important functions, exerted mainly through the action of adenosine on A1 receptors, concern the inhibition of lipolysis and the reduction of free fatty acids (whose involvement in the pathogenesis of insulin resistance, diabetes, cardiovascular diseases is recognized), the reduction in insulin resistance is associated with obesity, the increase in leptin production (with an additional beneficial effect on insulin sensitivity), and the increase in the uptake of glucose by adipocytes (with improvement of glucose tolerance but also in triglycerides storage and weigh gain). In agreement, in some studies on obese patients, A1Rs expression was found to be inversely related to the ability to lose weight (38, 39). As well as pyrimidine nucleotides, purine nucleotides positively affect innate immunity and regulates monocytes, macrophages, dendritic cells and mast-cell functions (37, 40). Moreover, their involvement in numerous central nervous system (CNS) functions such as behavior, nociception and locomotion has been highlighted (41).

Finally, the activation of purine pathway found in BM of overweight-obese mothers (15) could promote weight gain long after birth, but also improve glucose tolerance and insulin sensitivity in case of obesity, and reduce cardiovascular risk. It may also have a positive effect on the development of immune and anti-inflammatory processes and on neuroprotection.

## 5-methylthioadenosine

Methylthioadenosine (MTA) was increased 1 month post-partum in BM of obese-overweight mothers and it was also the only metabolite involved in the overlap between BMI and infant total fat content at 1 month (15). It is a natural nucleoside sulfur containing derived from 5 s-adenosylmethionine (5 SAMe) in polyamine cycle, present in all mammalian tissues including the placenta. 5 SAMe is the substrate of the 5-MTA phosphorylase enzyme, that initiates the salvage pathways leading to the recovery of methionine by one side and adenine (adenosine, AMP) by the other (39). It is an almost exclusively endogenous metabolite (its presence has been described but not quantified in some edible plants) described for the first time in BM by Isganaitis (15). Being a purine nucleoside, we can assume a gastro-intestinal absorption, similar to that of the other purines.

5-MTA has important cellular regulatory functions, including gene expression control, inhibition of cell proliferation, activation of lymphocytes, modulation of tumors invasiveness, regulation of apoptosis, and liver-protection (42). Its potent anti-inflammatory profile has been proven in mice where it prevents lipopolysaccharide-induced death by inhibiting TNFα production (pro-inflammatory cytokine) and iNOS **(**inducible nitric oxide synthase) gene expression, while enhancing the release of IL-10 (anti-inflammatory cytokine) (42).

In a metabolomics study on an induced rat model of diabetes dating back to 2016, 5 MTA was significantly higher. The study of oxidative stress products showed increased values of superoxide dismutase and hypoxia inducible factor 1 alpha. Both the 5-MAT and the oxidative stress products normalized after treatment with isoflavones, demonstrating their action on the cellular oxidative damage and therefore highlighting related metabolic processes (43). One year later, an untargeted metabolomics study (UPLC-MS) detected an increase in 5-MTA level in the urines of elderly patients with type 2 diabetes (T2D) (44). In a further metabolomics study in Mexican adolescents, conducted with to investigate the metabolites associated with a metabolic disease risk z-score (MetRisk z-score), 5-MTA values showed a significant correlation (45). Finally, 5-MTA levels resulted significantly associated with the BMI in a study conducted on 2,396 unrelated European individuals in the TwinsUK cohort and 724 others of the Health nucleus cohort in three time-points, covering a total interval of 8–18 years (46).

5-MTA should be considered a protective molecule against chronic inflammation and oxidative stress conditions characterizing obesity and metabolic syndrome. In this respect, MTA increase in BM of overweight-obese mothers (15) could be protective for long-term overweight, oxidative stress and cardio-metabolic risk.

## Sugar-Alcohols

Erythritol and ribitol (sugar alcohols) were increased in the milk of obese-overweight mothers 6 months after birth (15). To the best of our knowledge, only another study investigates sugar alcohols in BM (HPLC), even if erythritol and ribitol were not detectable (47). While ribitol is an endogenous molecule originating from the reduction of ribose in fibroblasts and erythrocytes, erythritol is a low-caloric sugar-substituted sweetener authorized in the USA and it is also present in different foods. It is absorbed from the proximal intestine by passive diffusion. Recently, its serum increase has been observed, in correlation to weight gain, in a cohort of freshmen. At the same time, through *ex vivo* isotopic techniques, endogenous production of erythritol from glucose (48) was detected. Given the wide use of low-caloric sweeteners by obese-overweight individuals, further investigation will be useful to clarify whether its increase in the milk of obese-overweight mothers could be related to this or to other metabolic pathways (48) and the possible long-term effect on glucose metabolism in the infant.

## Acylcarnitine and Amino Acids

*Acilcarnitine (ACs) and branched amino acids (BCAAs)*: Three short-chain ACs were increased in the milk of obese-overweight mothers at 6 months after delivery (15). Short-chain ACs derive from the catabolism of BCAAs, rather than from long-chain fatty acids. Although Isganaitis and colleagues did not detect an increase in BCAAs in the milk of overweight and obese mothers (15), De Luca et al. (16), trough UPLC and Tandem Mass (MS/MS), specifically measured free amino acids in the milk of 45 obese women and 45 controls at 1 month post-partum. As result, they found an increase in BCAAs and tyrosine (by 20 and 30%, respectively) in the formers (16). The increase in serum levels of BCAAs and ACs has been found, in several studies, in obese subjects with or without T2D, and has been more correlated with insulin resistance than with obesity (46, 49–52). The same group of Isganaitis (45) carried out an untargeted metabolomic study on 262 children aged 6–10 years divided into two groups (thin and obese non-diabetic): through MS, they found that obese children showed an increase in BCAAs and C3-C5 ACs, significantly correlated with several cardio-metabolic risk indices, including insulin resistance. Interestingly, the pattern was more pronounced in children of mothers with pre-gestational obesity.

An increase in BCAAs may compromise the transport of aromatic amino acids into cells and tissues, reducing the production of serotonin and melatonin (derived from tryptophan) and catecholamines (derived from phenylalanine and tyrosine) in the CNS. Melatonin and serotonin exert their effects both centrally and in the periphery, regulating energy homeostasis through central control of food intake, promoting lipogenesis and glucose metabolism (53).

Finally, literature data on BCAAs and ACs in obese subjects suggest that exposure to these metabolites is associated with increased metabolic risk later in life. It may also lead to a reduced availability of tryptophan and its serotonin and melatonin derivatives, with neurobehavioral impairment and negative effects on lipid metabolism. The abundance of BCAAs and ACs found in BM (15, 16) could in the same way be linked to an increased metabolic and neuropsychiatric risk in the long distance.

*Glutamine*: glutamine was reduced by about 30% in the milk of obese-overweight mothers at 6 months after delivery (15). It should be noted that an untargeted metabolomics work on the milk of mothers with gestational diabetes (54) showed a reduction of glutamine. Its role in promoting better glucose homeostasis as main precursor of gluconeogenesis in the kidney, representing a main substrate for gut-brain gluconeogenesis system, and an inducer of glucagon-like peptide secretion was highlighted in our recent review (11, 55–58) investigating BM of women with great obstetrical syndromes.

Finally, the reduction of glutamine in the milk of obese-overweight mothers at 6 months after birth (15) may have long-term adverse effects on glucose homeostasis.

*Asparagine and ornithine*: these aminoacids were reduced by about 50 and 20%, respectively, in the milk of obese-overweight mothers at 6 months after delivery (15). Several studies on the amino acid profile in obese subjects inversely correlate the amino acid asparagine with the cardio-metabolic risk (46, 50, 52, 59, 60); only one recent study, to our knowledge, correlates also ornithine to T2D (61).

Their reduction in the milk of obese women could be a long-term metabolic risk factor in infants.

*Aromatic amino acids and derivatives*: tyrosine (in addition to BCAAs) was increased by 30% in the milk of obese women 1 month after birth (16). An increase in blood tyrosine is frequently reported in obese subjects (45, 46, 50–52, 62, 63); it is likely to contribute to insulin resistance by glucose production through the pathway of dicarboxilic acid fumarate, the latter also found increased in BM of obese-overweight mothers at 6 months (15).

High levels of tyrosine in the milk of obese women (16) could have a negative impact on BMI and cardio-metabolic risk in later ages.

Kynuretic acid was reduced by about 30% in the milk of overweight-obese women 6 month after birth (15).

In the human body most of kynurenic acid comes from tryptophan catabolism; it is also present in foods and seems to be produced by intestinal microflora in moderate quantities. It can be easily absorbed from the digestive system and transported to the liver and the kidney (64).

In humans, serum kynurenic acid has been positively associated with several cardio-metabolic risk factors as BMI and insulin-resistance (35, 44): however, some reports in humans disagree with experimental studies where kynurenic acid was able to improve energy metabolism and inflammation in mice fed a high-fat diet and to promote weight loss (65, 66). Thus, it is possible that the elevation of this metabolite in individuals with metabolic risk can represent a compensatory mechanism through which kynurenic acid could perform its beneficial action. In this context, the reduction of kynurenic acid in BM of overweight-obese mothers in the Isganaitis study (15) could lead to an increased susceptibility to metabolic risk long after birth, rather than being protective.

*2-Aminobutyrate (2-AB):* 2-AB significantly increased in samples collected from overweight-obese womenat 1 month of lactation, increased by about 55% (*p* = 0.03) (15). 2-AB metabolismis poorly clarified, especially in BM. It seems involved in defense against oxidative stress; reduced glutathione is a central metabolite in the intracellular redox state. Glutathione consumption through oxidative stress activates a compensatory glutathione (GSH) synthetic pathway, accompanied by the synthesis of ophthalmic acid, a GSH analog, from 2-AB (67, 68).

We found a single study investigating 2-Ab in BM; in detail, such metabolite was compared between mothers affected by inflammatory bowel disease (IBD) and healthy mothers at 3 and 6 months of lactation. As result, 2-AB was lower in IBD mothers, potentially related to increased pro-inflammatory activity (69).

We strongly believe that 2-AB increase in BM from obese mothers could be a compensative mechanism reflecting an increase in oxidative stress.

## Polyamines

In the study by Ali et al. (17), 50 mothers (20 lean, 20 obese and 10 obese undergoing dietary treatment), were invited to collect milk at 3 days, 1 month, and 2 months post-partum, to investigate polyamine levels with HPLC. The total polyamine content was reduced in obese mothers at the three time points: the reduction concerned only spermidine and putrescine levels, while spermine was equally represented in the considered groups. The 10 obese mothers undergoing dietary treatment had a higher spermidine and putrescine milk content than the others obese women. The authors assume that the reduced content of polyamines in obese BM could depend on the low quantity of polyamines in the fat- and carbohydrates-rich foods assumed by obese subjects (17). In agreement, higher levels of polyamines were found in subjects who practice the Mediterranean diet (70).

Polyamines are widely present in the human body and are involved in many vital functions. Although they mainly derive from endogenous metabolism, a percentage is produced by food and intestinal flora, especially in case of fiber-rich diet. Once introduced with food, they can be absorbed and distributed to different tissues. Their protective role against cardio-metabolic risk has been highlighted by the study of Eisenberg et al., which finds an association between increased consumption of spermidine and decreased cardiovascular events and mortality (71).

Polyamines also represent oxidative stress and inflammation modulators, often associated with obesity and implicated in the pathogenesis of metabolic syndrome (72). In a study performed on 60 obese children aged 7–14 years, blood levels of polyamines were significantly higher than in controls, and spermine represented a marker of oxidative stress (NO pathway) and inflammation. The authors believe that the increase in these metabolites could be a protective mechanism against obesity-related oxidative stress (73). Recently, an association between serum polyamine and T2D levels has been reported in a cohort of patients with metabolic syndrome (74).

A deregulation of the polyamine system would play a role in neurodegenerative diseases (75, 76) and depression (77) because of their involvement in the modulation of synapsis and in the regulation of the ionic channels that participate in neuronal excitability (72).

Finally, the reduction of polyamines in BM could make newborns of obese mothers in the long distance more susceptible to weight gain, oxidative stress, inflammation and cardio-metabolic risk. Moreover, they may be less predisposed to develop neuropsychiatric disorders.

## Monosaccharides and Oligosaccharides

Below, we review the few available studies regarding human milk monosaccharides and oligosaccharides (HMOs) variations in BM from overweight and obese mothers instead of normal weight mothers' samples, and the consequent effects on neonatal metabolism and infant growth.

It should be underlined that ingested HMOs can reach the distal bowel and colon without undergoing any modification or enzymatic hydrolysis in the stomach and upper GI tract. Thus, HMOs can be metabolized by intestinal microbes and 99% of them is eliminated with the stools. The smallest percentage of them (about 1%) can be absorbed through the intestine and reach the circulation, being transferred to several organs, such as brain, liver, respiratory and urinary tract, where they can exert several functions (7).

- *1,5Anhydroglucitol (1,5-AG) (monosaccharide)* was found significantly increased in BM from overweight and obese mothers at 1 (*p* = 0.002)and 6 months of lactation (increased by 37% at 6 months, *p* = 0.003) (15).

1,5-AG is a very interesting metabolite not previously described in BM. Serum 1,5-AG is a validated short-term marker of glycemic control (78) in patients with type1 and type 2 diabetes, and its role in gestational diabetes is still under evaluation (78–82). During pregnancies affected by diabetes, the mean maternal serum values of 1,5-AG were negatively associated with neonatal birth weight and tended to be lower in infants with hypoglycemia; the magnitude of the difference between hypoglycemic and normoglycemic was greater for gestational diabetes (83).

According to another study, maternal serum level of 1,5-AG at birth was significantly and inversely associated with neonatal complications (such as respiratory distress, hypoglycemia, polycythemia, hyperbilirubinemia, and “large for gestational age” condition), resulting useful in the prediction of complications (84).

In our opinion, 1,5-AG level in BM could reflect maternal glycemic control and help in predicting neonatal outcome in pregnancies complicated by diabetes, even if it was not identified before in BM and its presence requires further clarification.

- *Arabinose (monosaccharide)* was found significantly increased in BM from overweight and obese mothers at 6 months of lactation (increased by 72%, *p* = 0.01) (15).

Arabinose, a five-carbon sugar is a carbon source for many bacteria. In literature, we did not find a metabolic role of such pentose in BM on infant weight gain.

However, in two studies it seems to modulate, in a controversial way, *Pseudomonas aeruginosa* virulence, an opportunistic human pathogen strongly associated with NEC development (85, 86).

Therefore, arabinose could have a potential role on pathogens virulence, improving our knowledge of BM-related effects and contributing to the optimization of formula milks.

- *Glucose-6-phosphate (monosaccharide)* was significantly higher in BM from overweight and obese mothers at 6 months of lactation (2.07-fold change, *p* = 0.01) (15).

Glucose-6-phosphate (G6P or α-D-glucose-6-phosphate) is involved in protection against oxidative stress, since it guarantees adequate levels of NADPH to modulate the redox state (87).

In literature, we found a single very old study measuring such metabolite in human milk during established times of lactation, including G6P, but the results are not clear (88).

Thus, by the few available evidences, it could be supposed a protective role against oxidative stress and provide energy supply.

- *Lacto-N-fucopentaose I (LNFPI)* was found significantly reduced in BM from overweight and obese mothersat 1 month of lactation, reduced by about 62% compared to samples of normal weight mothers (*p* = 0.007) (15).

LNFPI resulted the most relevant influencer of infant growth, significantly associated with lower infant weight at 1 month and with lower weight and less lean and fat mass at 6 months (89).

LNFPI was also associated with lower infant weight and weight gain at 1 month in another study (18).

Moreover, LNFPI had positive contributions in height-for-age Z scores at 20 weeks (30).

LFNP I was lower in BM of mothers of severely stunted infants vs. healthy controls 6 months after delivery (90).

Therefore, LNFPI in BM seems protective against excessive weight gain in infants and its reduction in overweight and obesity supports the potential pro-adipogenic role of BM in these mothers.

- *Lacto-N-fucopentaose II and III (LNFPII-III)* were found significantly higher in BM from overweight and obese mothers at 1 month of lactation (*p* < 0.05) (15).

At 6 months of lactation, LNFPII was associated with greater fat mass (89).

BM LNFP III positively contributed to infant height-for-age Z scores at 20 weeks (30) and it seems to modulate metabolic pathways in mice, improving glucose tolerance, insulin sensitivity and suppressing liver lipogenesis in experimental model of obesity (91).

In conclusion, little is known on such HMOs and their impact on neonatal growth; LNFP II high level in BM of overweight and obese mothers could suggest a role in infant overweight.

- *2-Fucosyllactose (2*′*-FL)* was found significantly lower in BM from overweight and obese mothers at 1 month of lactation, reduced by about 38% than normal weight mothers samples (15).

On the contrary, in another study, 2′-FL would be higher in samples from overweight than non overweight mothers (18).

Regarding its relation to infant growth, 2′-FL was found lower in BM of severely stunted infants' mothers vs. healthy controls at 6 months of life (90).

2′-FL seems directly associated to maternal pre-pregnancy BMI, infant weight up to 1 year of life and also child height up to 5 years of life in offspring of *Se*+ mothers. According to such data, this HMOs seems to affect child growth up to 5 years of life (92).

Contrarily, in another study 2′-FL did not influence body length, weight, CC or BMI at 4 months of life (93).

In milk from Se+ mothers, 2′-FL resulted the most abundant HMO, positively associated with infant weight velocity from 0 to 5 months of post-natal age and with fat mass index at 5 months. Thus, 2-FL, currently added to some milk formulas, could be involved in excessive weight gain (94).

Maternal BMI at 5 months of lactation has been positively correlated with 2′-FL content (94).

In conclusion, data on 2′-FL seems conflicting; such HMO was associated with a higher maternal BMI and with infant growth, even if its variation in BM from obese mothers should be still defined.

2′-FLwas also hypothesized to increase weight gain when milk formulas are supplemented, effect that results balanced by other HMOs added to such formulations (95, 96).

- *3-Fucosyllactose (3*′*-FL)* was found significantly increased in BM from overweight and obese mothers at 1 month of lactation (*p* = 0.03) (15).

Contrarily, in another study, 3′-FL was lower in BM from *Se*+ overweight mothers than normal weigh ones (18).

Regarding 3′-FL content in BM of overweight and obese mothers, data are controversial and there are not significant associations with infant growth.

- *Lacto-N-hexaose (LNH)* was found significantly lower in BM of obese mothers at 3–4 months of lactation (*p* < 0.05) (19).

At 6 months of lactation, it has been associated with higher infants' body fat mass (89); thus, LNH reduction could protect from weight gain.

Moreover, it could contribute to the lower duration of breastfeeding of obese mothers (18, 19).

## Lipids

Lipid composition of BM seems influenced by maternal BMI, influencing its inflammatory and oxidative profile (20).

Maternal obesity seems related to the increase of n-6/n-3 fatty acid ratio and to the proportion of long-chain fatty acids (LCFAs) in BM. (96, 21–24). LCFAs are less digestible by the neonatal gut; moreover, long-chain polyunsaturated fatty acids (LCPUFAs), such as eicosapentaenoic acid (EPA), docosahexaenoic acid (DHA), and arachidonic acid (AA) are involved in several metabolic pathways, including a role as constituents of cells membranes, and in nervous system and retina development (20).

Infant growth up to 6 months of life was correlated with higher levels of dihomo-gamma-linolenic (20:3 n6-DGLA), adrenic (22:4 n-6), palmitic acids, conjugated linoleic isomers and reduced level of oleic acids in BM of overweight-obese mothers (25).

Palmitic acid seems involved in inflammatory and metabolic responses and it could also reduce the oxidation of FAs, alter the insulin response and increase the fat mass (97).

Adrenic acid is abundant in the brain and in myelin lipids, especially in phosphatidylethanolamine. Its important precursor is AA acid, whose conversion into adrenic acid is particularly active in the early stages of life (26). Oleic acid was shown to reduce obesity risk and improve the metabolic and lipid profiles in adults (98). DGLA, other n-6 PUFAs such as AA, and bosseopentaenoic acid (20:5 n-6) are probably involved in the pathogenesis of obesity, promoting adipogenesis and inflammation (20). This group correlates with a greater increase in the weight for age (WFA) z-score, a smaller increase in the lengh for age z-score and of the CC between 2 weeks and 2 months of age. The effects on long distance obesity are clearly to be determined (25).

In another study, samples from overweight mothers showed higher levels of saturated FAs (SFAs), lower amount of n-3 FAs and lower ratio of unsaturated (UFA) to saturated (SFA) FAs, and higher n-6/n-3ratio than normal weight samples. Moreover, total SFAs content in BM was positively correlated while MUFA/SFA ratio and UFA/SFA ratio inversely correlated to infant weight and BMI gain up to 13 months (21).

Successively, the same group investigated the combined effects of maternal pre-pregnancy BMI and food choices on BM triacylglycerols (TAGs) at 3 months of lactation. They evidenced a higher content in 18:3 and a reduced level of 18:0 in normal weight mothers following a recommended food-diet (low fat), than normal weight mothers eating non-recommended foods. Moreover, in samples collected from normal weight mothers eating recommended foods, levels of 50:1 were lower than milk produced by overweight mothers eating recommended food choices. Finally, BM from overweight and obese mothers was characterized by higher levels of saturated FA and lower amount of n-3 FA than non overweight mothers, independently by the diet. Thus, they concluded that maternal BMI and diet can influence the molecular weight distribution of TAGs in BM samples but does not significantly alter their regioisomerism (27).

In BM from obese mothers, an increase in lipid content (10–20%), and higher levels of ALA, n-6/ n-3 ratio and total PUFA were detect, instead of normal weight mothers. In the same study, total MUFAwere significantly reduced in BM from overweight and obese mothers, while 20:1 n-9 were increased (22).

In a similar article,n-3 LCPUFA (including EPA and DHA) were lower while n-6 LCPUFA and n-6/n-3 ratio were higher in overweight mothers at 6–7 months of lactation (20).

A Sweden group demonstrated higher SFAs and n-6/n-3/ratio, and lower n-3LCPUFA (and LA, DHA, EPA) in BM of obese mothers (24).

Total PAHSA levels [the fatty acid esters of hydroxy fatty acids (FAHFAs), namely palmitic acid hydroxystearic acids], endogenous lipid produced by adipocytes in the mammary gland, resulted significantly lower in obese mothers' samples at 3 days postpartum (28).

PAHSA seem to promote gut maturation and secretion of GLP-1, which stimulates insulin secretion and increase glucose tolerance (99).

Interesting findings were also obtained correlating maternal BM lipid content in colostrum and mature milk with infant anthropometry (at 6, 18, and 36 months) and with cognition at 18 months. BM from overweight and obese mothers showed higher SFA levels and n-6/n-3 ratio, and decreased ALA, DHA and MUFA content in mature milk. Infant BMI-z-score at 6 months resulted inversely associated with colostrum levels of n-6 and n-3 LC-PUFAs (e.g., AA and DHA) and positively associated with n-6/n-3 ratio. Cognitive profile evaluated with Bayley scales was positively correlated to colostrum content of n-6 and n-3 PUFAs, DHA, and ALA, and negative correlated to the n-6/n-3 ratio. Thus, according to these data, maternal obesity could increase BMI in the offspring, but n-6/n-3 ratio could impair infant cognition, even if such results should be confirmed (23).

In a further study BM of obese mothers at 2 months post-partum showed a higher n-6/n-3 FA ratio, while total n-3 PUFAs were reduced of 20%, in association to lower levels of DHA, EPA, docosapentaenoic acid and lutein (29).

A unique study of Sinanoglu et al. deeply investigated colostrum lipid content according to maternal BMI. As result, docosadienoic acid (C22:2 n-6), conjugated LA isomers C18:2*c*9*t*11 (rumenic acid) and C18:2*t*11*t*13 were higher in normal weight mothers' BM; total n-3,decanoic (C10:1), tridecanoic (C13:0) and adrenic (C22:4n-6) acids were higher in obese mothers; ALA (C18:3n-3) was higher in overweight mothers' samples (26).

Interest in conjugated LA isomers has grown in recent years due to the increasing number of experimental studies attributing them anti-inflammatory, anti-carcinogenic, antiadipogenic, antidiabetic, and anti-hypertensive properties in animal models. Their increase is probably causedby the excessive dietary intake (25). They may represent afuture nutritional tool to prevent diseases as metabolic syndrome but studies on humans are still necessary (100–102).

The n-6/n-3 ratio in BM increases in proportion with the fat in the diet: this could lead to a higher adipose tissue accumulation in neonates fed with obese mothers' BM. The authors speculate that the increase of PUFA in overweight and obese mothers' BM could determine cardio-protective (probably) compensatory mechanism for their infants, since such mediators are related to a better metabolic profile (26).

## Conclusions

Maternal obesity seems a major risk factor for excessive fetal growth (103), infant overweight and children obesity (104, 105). The negative impact of obesity on children's health can lead to the early development of T2D, the premature onset of cardiovascular complications and, in general, a higher risk of early mortality (106). Recent literature has shown increasing interest in the impact of such disease on the nervous system and in particular, in the field of neuropsychiatric and behavioral disorders (mainly attention-deficit/hyperactivity disorder, conduct disorders and autism), described only sporadically in the past but now confirmed by large population studies (107, 108). Besides, a reduction in prefrontal cortex thickness and associated executive function deficit was described in a large cohort of obese children aged 9–10 years (109).

In the present review of metabolomics studies on BM from overweight-obese mothers highlighting the differences with samples from the lean ones, some of the metabolites that differentiate the two groups—aminoacids, acyl-carnitines, lipids and oligosaccharides—have been found altered in subjects with the same characteristics and in experimental modes of obesity in several studies and in a relevant number of cases. For these metabolites, which can be considered “major,” the results obtained from the aforementioned studies allow for more reliable hypotheses on the meaning of their alteration in BM long after birth. However, it should be noted that it is difficult to make qualitative assessments of the relevance of one group of metabolites in relation to the other.

Between these “major” metabolites, all aminoacids found altered could promote cardio-metabolic risk unlike their derivatives (“minor” metabolites) whose reduction (kinurenic acid) or increase (2-aminobutyrate) might give protection against cardio-metabolic risk from oxidative stress and inflammation.

Among HMOs, those decreased (LNFPI, 2FL, LNH) seem to have a protective effect against excessive weight gain, while NFPII increase could predispose to it.

Our analysis of the papers investigating fatty acids in BM of overweight-obese mothers, in agreement with the unique available meta-analysis (110), highlights an increase in saturated fatty acids, a reduction in monounsaturated fatty acids, a reduction in n-3 LCPUFA, and an increase in n-6/n-3 ratio in most of them. The first two effects could promote excessive long-term weight gain and associated inflammation, as well as reduce glucose tolerance. The increase in the n-6/n-3 ratio, due to the reduction in n-3 or increase in n-6 may impair, as previously mentioned, sensorineural development and promote adipogenesis and inflammation.

Between the “minor” metabolites, the alteration of nucleotides derivatives could promote weight gain after birth and have, except for adenine and MTA, a negative impact on the risk of insulin resistance, T2D, cardio-metabolic disease and on the onset of neurological problems. Increase of adenine, while promoting weight gain, could hinder the insulin resistance associated with obesity (and cardio-vascular risk) and have a positive impact on immune and neurological development. Even MTA increase could be protective against cardio-metabolic risk in offspring.

Lastly, reduction of polyamines could correlate with outcomes related to obesity in the long distance.

Finally, metabolites variations in BM samples collected from overweight-obese mothers and the potentially correlated effects pointed out above, still need further investigations and should be confirmed in future studies on larger sample: metabolomics seems the most suitable technology in this regard. It can lead to the comprehensive description of such biofluid and the related effects on breastfed subjects, potentially highlighting personalized needs of BM supplementation or short and long-term prevention strategies to optimize offspring health.

In our opinion, such topic is of major importance, since pediatric health starts in intrauterine and perinatal life.

## Author Contributions

VF conceptualized the paper. FB and MP updated the literature and wrote the first version of the review. VF and DP critically revised the text. All authors contributed to the article and approved the submitted version.

## Conflict of Interest

The authors declare that the research was conducted in the absence of any commercial or financial relationships that could be construed as a potential conflict of interest.

## References

[1] WHO technical staff Exclusive Breastfeeding to Reduce the Risk of Childhood Overweight and Obesity. Biological, Behavioural and Contextual Rationale. Geneva: e-Library of Evidence for Nutrition Actions (eLENA) (2014).

[2] Oken E Fields DA Lovelady CA Redman LM. TOS scientific position statement: breastfeeding and obesity. Obesity. (2017) 25:1864–6 10.1002/oby.22024 29086503 PMC9048856

[3] Woo JG Martin LJ. Does breastfeeding protect against childhood obesity? Moving beyond observational evidence. Curr Obes Rep. (2015) 4:207–16. 10.1007/s13679-015-0148-9 26627216

[4] Li C Kaur H Choi WS Huang TT Lee RE Ahluwalia JS. Additive interactions of maternal prepregnancy BMI and breast-feeding on childhood overweight. Obes Res. (2005) 13:362–71. 10.1038/oby.2005.48 15800295

[5] Bardanzellu F Fanos V Reali A. “Omics” in human colostrum and mature milk: looking to old data with new eyes. Nutrients. (2017) 9:843. 10.3390/nu9080843 28783113 PMC5579636

[6] Bardanzellu F Fanos V Strigini FAL Artini PG Peroni DG. Human breast milk: exploring the linking ring among emerging components. Front Pediatr. (2018) 6:215. 10.3389/fped.2018.00215 30131948 PMC6091001

[7] Bardanzellu F Reali A Marcialis MA Fanos V. Across the “sweetest” properties of human breast milk: focus on oligosaccharides. Curr Pediatr Rev. (2020). 10.2174/1573396316666200429114217. [Epub ahead of print]. 32348229

[8] Bardanzellu F Peroni DG Fanos V. Human breast milk: bioactive components, from stem cells to health outcomes. Curr Nutr Rep. (2020) 9:1–13. 10.1007/s13668-020-00303-7 31927722

[9] Bardanzellu F Peila C Fanos V Coscia A. Clinical insights gained through metabolomic analysis of human breast milk. Expert Rev Proteomics. (2019) 16:909–32. 10.1080/14789450.2019.1703679 31825672

[10] Congiu M Reali A Deidda F Dessì A Bardanzellu F Fanos V. Breast milk for preterm multiples: more proteins, less lactose. Twin Res Hum Genet. (2019) 22:265–71. 10.1017/thg.2019.42 31337466

[11] Bardanzellu F Puddu M Fanos V. The human breast milk metabolome in preeclampsia, gestational diabetes, and intrauterine growth restriction: implications for child growth and development. J Pediatr. (2020) 221S:S20–8. 10.1016/j.jpeds.2020.01.049 32482230

[12] Marincola FC Noto A Caboni P Reali A Barberini L Lussu M. A metabolomic study of preterm human and formula milk by high resolution NMR and GC/MS analysis: preliminary results. J Matern Fetal Neonatal Med. (2012) 25(Suppl. 5):62–7. 10.3109/14767058.2012.715436 23025771

[13] Cesare Marincola F Dessì A Corbu S Reali A Fanos V. Clinical impact of human breast milk metabolomics. Clin Chim Acta. (2015) 451(Pt A):103–6. 10.1016/j.cca.2015.02.021 25689794

[14] Sundekilde UK Downey E O'Mahony JA O'Shea CA Ryan CA Kelly AL. The effect of gestational and lactational age on the human milk metabolome. Nutrients. (2016) 8:304. 10.3390/nu8050304 27213440 PMC4882716

[15] Isganaitis E Venditti S Matthews TJ Lerin C Demerath EW Fields DA. Maternal obesity and the human milk metabolome: associations with infant body composition and postnatal weight gain. Am J Clin Nutr. (2019) 110:111–20. 10.1093/ajcn/nqy334 30968129 PMC6599743

[16] De Luca A Hankard R Alexandre-Gouabau MC Ferchaud-Roucher V Darmaun D Boquien CY. Higher concentrations of branched-chain amino acids in breast milk of obese mothers. Nutrition. (2016) 32:1295–8. 10.1016/j.nut.2016.05.013 27497516

[17] Ali MA Strandvik B Palme-Kilander C Yngve A. Lower polyamine levels in breast milk of obese mothers compared to mothers with normal body weight. J Hum Nutr Diet. (2013) 26(Suppl. 1):164–70. 10.1111/jhn.12097 23627874

[18] Tonon KM de Morais MB Abrão ACFV Miranda A Morais TB Maternal and infant factors associated with human milk oligosaccharides concentrations according to secretor and lewis phenotypes. Nutrients. (2019) 11:1358 10.3390/nu11061358 PMC6628139 31212920

[19] Azad MB Robertson B Atakora F Becker AB Subbarao P Moraes TJ. Human milk oligosaccharide concentrations are associated with multiple fixed and modifiable maternal characteristics, environmental factors, and feeding practices. J Nutr. (2018) 148:1733–42. 10.1093/jn/nxy175 30247646

[20] García-Ravelo S Díaz-Gómez NM Martín MV Dorta-Guerra R Murray M Escuder D. Fatty acid composition and eicosanoid levels (LTE_4_ and PGE_2_) of human milk from normal weight and overweight mothers. Breastfeed Med. (2018) 13:702–10. 10.1089/bfm.2017.0214 30325649

[21] Mäkelä J Linderborg K Niinikoski H Yang B Lagström H. Breast milk fatty acid composition differs between overweight and normal weight women: the STEPS Study. Eur J Nutr. (2013) 52:727–35. 10.1007/s00394-012-0378-5 22639073

[22] Marín MC Sanjurjo A Rodrigo MA de Alaniz MJ. Long-chain polyunsaturated fatty acids in breast milk in La Plata, Argentina: relationship with maternal nutritional status. Prostaglandins Leukot Essent Fatty Acids. (2005) 73:355–60. 10.1016/j.plefa.2005.07.005 16129590

[23] de la Garza Puentes A MartíAlemany A Chisaguano AM Montes Goyanes R Castellote AI Torres-Espínola FJ. The effect of maternal obesity on breast milk fatty acids and its association with infant growth and cognition-the PREOBE follow-up. Nutrients. (2019) 11:2154. 10.3390/nu11092154 31505767 PMC6770754

[24] StorckLindholm E Strandvik B Altman D Möller A Palme Kilander C. Different fatty acid pattern in breast milk of obese compared to normal-weight mothers. Prostaglandins Leukot Essent Fatty Acids. (2013) 88:211–7. 10.1016/j.plefa.2012.11.007 23273824

[25] Ellsworth L Perng W Harman E Das A Pennathur S Gregg B. Impact of maternal overweight and obesity on milk composition and infant growth. Matern Child Nutr. (2020) 19:e12979. 10.1111/mcn.12979 32074402 PMC7296794

[26] Sinanoglou VJ Cavouras D Boutsikou T Briana DD Lantzouraki DZ Paliatsiou S. Factors affecting human colostrum fatty acid profile: a case study. PLoS ONE. (2017) 12:e0175817. 10.1371/journal.pone.0175817 28410426 PMC5391953

[27] Linderborg KM Kalpio M Mäkelä J Niinikoski H Kallio HP Lagström H. Tandem mass spectrometric analysis of human milk triacylglycerols from normal weight and overweight mothers on different diets. Food Chem. (2014) 146:583–90. 10.1016/j.foodchem.2013.09.092 24176384

[28] Brezinova M Kuda O Hansikova J Rombaldova M Balas L Bardova K. Levels of palmitic acid ester of hydroxystearic acid (PAHSA) are reduced in the breast milk of obese mothers. Biochim Biophys Acta Mol Cell Biol Lipids. (2018) 1863:126–31. 10.1016/j.bbalip.2017.11.004 29154942

[29] Panagos PG Vishwanathan R Penfield-Cyr A Matthan NR Shivappa N Wirth MD. Breastmilk from obese mothers has pro-inflammatory properties and decreased neuroprotective factors. J Perinatol. (2016) 36:284–90. 10.1038/jp.2015.199 26741571 PMC4888773

[30] Davis JC Lewis ZT Krishnan S Bernstein RM Moore SE Prentice AM. Growth and morbidity of gambian infants are influenced by maternal milk oligosaccharides and infant gut microbiota. Sci Rep. (2017) 7:40466. 10.1038/srep40466 28079170 PMC5227965

[31] Schlimme E Martin D Meisel H. Nucleosides and nucleotides: natural bioactive substances in milk and colostrum. Br J Nutr. (2000) 84 (Suppl 1):S59–68. 10.1017/S0007114500002269 11242448

[32] Löffler M Fairbanks LD Zameitat E Marinaki AM Simmonds HA. Pyrimidine pathways in health and disease. Trends Mol Med. (2005) 11:430–7. 10.1016/j.molmed.2005.07.003 16098809

[33] Löffler M Carrey EA Zameitat E. Orotic acid, more than just an intermediate of pyrimidine *de novo* synthesis. J Genet Genomics. (2015) 42:207–19. 10.1016/j.jgg.2015.04.001 26059769

[34] Lazarowski ER Harden TK. UDP-sugars as extracellular signaling molecules: cellular and physiologic consequences of P2Y14 receptor activation. Mol Pharmacol. (2015) 88:151–60. 10.1124/mol.115.098756 25829059 PMC4468635

[35] Meister J Le Duc D Ricken A Burkhardt R Thiery J Pfannkuche H. The G protein-coupled receptor P2Y14 influences insulin release and smooth muscle function in mice. J Biol Chem. (2014) 289:23353–66. 10.1074/jbc.M114.580803 24993824 PMC4156089

[36] Gessi S Merighi S Fazzi D Stefanelli A Varani K Borea PA. Adenosine receptor targeting in health and disease. Exp Opin Investig Drugs. (2011) 20:1591–609. 10.1517/13543784.2011.627853 22017198

[37] Tozzi M Novak I. Purinergic receptors in adipose tissue as potential targets in metabolic disorders. Front Pharmacol. (2017) 8:878. 10.3389/fphar.2017.00878 29249968 PMC5715378

[38] Barakat H Davis J Lang D Mustafa SJ McConnaughey MM. Differences in the expression of the adenosine A1 receptor in adipose tissue of obese black and white women. J Clin Endocrinol Metab. (2006) 91:1882–6. 10.1210/jc.2005-2109 16507638

[39] Johnson JA Fried SK Pi-Sunyer FX Albu JB. Impaired insulin action in subcutaneous adipocytes from women with visceral obesity. Am J Physiol Endocrinol Metab. (2001) 280:E40–9. 10.1152/ajpendo.2001.280.1.E40 11120657

[40] Dhalla AK Chisholm JW Reaven GM Belardinelli L. A1 adenosine receptor: role in diabetes and obesity. Handb Exp Pharmacol. (2009) 193:271–95. 10.1007/978-3-540-89615-9_9 19639285

[41] Borea PA Varani K Vincenzi F Baraldi PG Tabrizi MA Merighi S. The A3 adenosine receptor: history and perspectives. Pharmacol Rev. (2015) 67:74–102. 10.1124/pr.113.008540 25387804

[42] Avila MA García-Trevijano ER Lu SC Corrales FJ Mato JM. Methylthioadenosine. Int J Biochem Cell Biol. (2004) 36:2125–30. 10.1016/j.biocel.2003.11.016 15313459

[43] Zhang Y Wang P Xu Y Meng X Zhang Y. Metabolomic analysis of biochemical changes in the plasma of high-fat diet and streptozotocin-induced diabetic rats after treatment with isoflavones extract of radix puerariae. Evid Based Comp Altern Med. (2016) 2016:4701890. 10.1155/2016/4701890 27042190 PMC4794592

[44] Tam ZY Ng SP Tan LQ Lin CH Rothenbacher D Klenk J. Metabolite profiling in identifying metabolic biomarkers in older people with late-onset type 2 diabetes mellitus. Sci Rep. (2017) 7:4392. 10.1038/s41598-017-01735-y 28663594 PMC5491522

[45] Perng W Hector EC Song PXK Tellez Rojo MM Raskind S Kachman M. Metabolomic determinants of metabolic risk in mexican adolescents. Obesity. (2017) 25:1594–602. 10.1002/oby.21926 28758362 PMC5573626

[46] Cirulli ET Guo L Leon Swisher C Shah N Huang L Napier LA. Profound perturbation of the metabolome in obesity is associated with health risk. Cell Metab. (2019) 29:488–500.e2. 10.1016/j.cmet.2018.09.022 30318341 PMC6370944

[47] Cavalli C Teng C Battaglia FC Bevilacqua G. Free sugar and sugar alcohol concentrations in human breast milk. J Pediatr Gastroenterol Nutr. (2006) 42:215–21. 10.1097/01.mpg.0000189341.38634.77 16456418

[48] Hootman KC Trezzi JP Kraemer L Burwell LS Dong X Guertin KA. Erythritol is a pentose-phosphate pathway metabolite and associated with adiposity gain in young adults. Proc Natl Acad Sci USA. (2017) 114:E4233–40. 10.1073/pnas.1620079114 28484010 PMC5448202

[49] Schooneman MG Vaz FM Houten SM Soeters MR Acylcarnitines: reflecting or inflicting insulin resistance? Diabetes. (2013) 62:1–8. 10.2337/db12-0466 23258903 PMC3526046

[50] Cheng S Rhee EP Larson MG Lewis GD McCabe EL Shen D. Metabolite profiling identifies pathways associated with metabolic risk in humans. Circulation. (2012) 125:2222–31. 10.1161/CIRCULATIONAHA.111.067827 22496159 PMC3376658

[51] Newgard CB An J Bain JR Muehlbauer MJ Stevens RD Lien LF. A branched-chain amino acid-related metabolic signature that differentiates obese and lean humans and contributes to insulin resistance. Cell Metab. (2009) 9:311–26. 10.1016/j.cmet.2009.02.002 19356713 PMC3640280

[52] Libert DM Nowacki AS Natowicz MR. Metabolomic analysis of obesity, metabolic syndrome, and type 2 diabetes: amino acid and acylcarnitine levels change along a spectrum of metabolic wellness. PeerJ. (2018) 6:e5410. 10.7717/peerj.5410 30186675 PMC6120443

[53] Höglund E Øverli Ø Winberg S. Tryptophan metabolic pathways and brain serotonergic activity: a comparative review. Front Endocrinol. (2019) 10:158. 10.3389/fendo.2019.00158 31024440 PMC6463810

[54] Chen Q Francis E Hu G Chen L. Metabolomic profiling of women with gestational diabetes mellitus and their offspring: review of metabolomics studies. J Diabetes Comp. (2018) 32:512–23. 10.1016/j.jdiacomp.2018.01.007 29506818

[55] Darmaun D Torres-Santiago L Mauras N. Glutamine and type 1 diabetes mellitus: is there a role in glycemic control? Curr Opin Clin Nutr Metab Care. (2019) 22:91–5. 10.1097/MCO.0000000000000530 30461450

[56] Soty M Gautier-Stein A Rajas F Mithieux G. Gut-brain glucose signaling in energy homeostasis. Cell Metab. (2017) 25:1231–42. 10.1016/j.cmet.2017.04.032 28591631

[57] Doyle ME Egan JM. Mechanisms of action of glucagon-like peptide 1 in the pancreas. Pharmacol Ther. (2007) 113:546–93. 10.1016/j.pharmthera.2006.11.007 17306374 PMC1934514

[58] Hassel B Dingledine R Glutamate. In: Brady SR, Siegel J, Albers RW, Price DL, editors. Basic Neurochemistry. Amsterdam: Academic Press (2012). p. 342–66.

[59] Ottosson F Smith E Melander O Fernandez C. Altered asparagine and glutamate homeostasis precede coronary artery disease and type 2 diabetes. J Clin Endocrinol Metab. (2018) 103:3060–9. 10.1210/jc.2018-00546 29788285

[60] Takashina C Tsujino I Watanabe T Sakaue S Ikeda D Yamada A. Associations among the plasma amino acid profile, obesity, and glucose metabolism in Japanese adults with normal glucose tolerance. Nutr Metab. (2016) 13:5. 10.1186/s12986-015-0059-5 26788116 PMC4717594

[61] Cao YF Li J Zhang Z Liu J Sun XY Feng XF. Plasma levels of amino acids related to urea cycle and risk of type 2 diabetes mellitus in chinese adults. Front Endocrinol. (2019) 10:50. 10.3389/fendo.2019.00050 30833930 PMC6387924

[62] Hellmuth C Kirchberg FF Lass N Harder U Peissner W Koletzko B. Tyrosine is associated with insulin resistance in longitudinal metabolomic profiling of obese children. J Diabetes Res. (2016) 2016:2108909. 10.1155/2016/2108909 26881241 PMC4736430

[63] Butte NF Liu Y Zakeri IF Mohney RP Mehta N Voruganti VS. Global metabolomic profiling targeting childhood obesity in the Hispanic population. Am J Clin Nutr. (2015) 102:256–67. 10.3945/ajcn.115.111872 26085512 PMC4515872

[64] Wirthgen E Hoeflich A Rebl A Günther J. Kynurenic acid: the janus-faced role of an immunomodulatory tryptophan metabolite and its link to pathological conditions. Front Immunol. (2018) 8:1957. 10.3389/fimmu.2017.01957 29379504 PMC5770815

[65] Agudelo LZ Ferreira DMS Cervenka I Bryzgalova G Dadvar S Jannig PR. Kynurenic acid and Gpr35 regulate adipose tissue energy homeostasis and inflammation. Cell Metab. (2018) 27:378–92.e5. 10.1016/j.cmet.2018.01.004 29414686

[66] Dadvar S Ferreira DMS Cervenka I Ruas JL. The weight of nutrients: kynurenine metabolites in obesity and exercise. J Intern Med. (2018) 284:519–33. 10.1111/joim.12830 30141532

[67] Irino Y Toh R Nagao M Mori T Honjo T Shinohara M. 2-Aminobutyric acid modulates glutathione homeostasis in the myocardium. Sci Rep. (2016) 6:36749. 10.1038/srep36749 27827456 PMC5101505

[68] Ghosh S Forney LA Wanders D Stone KP Gettys TW. An integrative analysis of tissue-specific transcriptomic and metabolomic responses to short-term dietary methionine restriction in mice. PLoS ONE. (2017) 12:e0177513. 10.1371/journal.pone.0177513 28520765 PMC5433721

[69] Meng X Dunsmore G Koleva P Elloumi Y Wu RY Sutton RT. The profile of human milk metabolome, cytokines, and antibodies in inflammatory bowel diseases versus healthy mothers, and potential impact on the newborn. J Crohns Colitis. (2019) 13:431–41. 10.1093/ecco-jcc/jjy186 30418545 PMC6441305

[70] Ramos-Molina B Queipo-Ortuño MI Lambertos A Tinahones FJ Peñafiel R. Dietary and gut microbiota polyamines in obesity- and age-related diseases. Front Nutr. (2019) 6:24. 10.3389/fnut.2019.00024 30923709 PMC6426781

[71] Eisenberg T Abdellatif M Schroeder S Primessnig U Stekovic S Pendl T. Cardioprotection and lifespan extension by the natural polyamine spermidine. Nat Med. (2016) 22:1428–38. 10.1038/nm.4222 27841876 PMC5806691

[72] Pegg AE Functions of polyamines in mammals. J Biol Chem. (2016) 291:14904–12. 10.1074/jbc.R116.731661 27268251 PMC4946908

[73] Codoñer-Franch P Tavárez-Alonso S Murria-Estal R Herrera-Martín G Alonso-Iglesias E. Polyamines are increased in obese children and are related to markers of oxidative/nitrosative stress and angiogenesis. J Clin Endocrinol Metab. (2011) 96:2821–5. 10.1210/jc.2011-0531 21697248

[74] Fernandez-Garcia JC Delpino-Rius A Samarra I Castellano-Castillo D Muñoz-Garach A Bernal-Lopez MR. Type 2 diabetes is associated with a different pattern of serum polyamines: a case-control study from the PREDIMED-plus trial. J Clin Med. (2019) 8:71. 10.3390/jcm8010071 30634588 PMC6352090

[75] Joaquim HPG Costa AC Forlenza OV Gattaz WF Talib LL Decreased plasmatic spermidine and increased spermine in mild cognitive impairment and Alzheimer's disease patients. Arch Clin Psychiatry. (2019) 46:120–4. 10.1590/0101-60830000000209

[76] Lewandowski NM Ju S Verbitsky M Ross B Geddie ML Rockenstein E. Polyamine pathway contributes to the pathogenesis of Parkinson disease. Proc Natl Acad Sci USA. (2010) 107:16970–5. 10.1073/pnas.1011751107 20837543 PMC2947879

[77] Limon A Mamdani F Hjelm BE Vawter MP Sequeira A. Targets of polyamine dysregulation in major depression and suicide: activity-dependent feedback, excitability, and neurotransmission. Neurosci Biobehav Rev. (2016) 66:80–91. 10.1016/j.neubiorev.2016.04.010 27108532 PMC5096383

[78] Dungan KM. 1,5-anhydroglucitol (GlycoMark) as a marker of short-term glycemic control and glycemic excursions. Exp Rev Mol Diagn. (2008) 8:9–19. 10.1586/14737159.8.1.9 18088226

[79] Pramodkumar TA Jayashri R Gokulakrishnan K Velmurugan K Pradeepa R Venkatesan U. 1,5 Anhydroglucitol in gestational diabetes mellitus. J Diabetes Comp. (2019) 33:231–5. 10.1016/j.jdiacomp.2018.11.010 30594413

[80] Kim WJ Park CY. 1,5-Anhydroglucitol in diabetes mellitus. Endocrine. (2013) 43:33–40. 10.1007/s12020-012-9760-6 22847316

[81] Ikeda N Hara H Hiroi Y Nakamura M. Impact of serum 1,5-anhydro-d-glucitol level on prediction of major adverse cardiac and cerebrovascular events in non-diabetic patients without coronary artery disease. Atherosclerosis. (2016) 253:1–6. 10.1016/j.atherosclerosis.2016.08.016 27569457

[82] Ikeda N Hiroi Y Cardiovascular disease and 1,5-anhydro-d-glucitol. Global Health Med. (2019) 1:83–7. 10.35772/ghm.2019.01031 PMC7731190 33330760

[83] Wright LA Hirsch IB Gooley TA Brown Z. 1,5-anhydroglucitol and neonatal complications in pregnancy complicated by diabetes. Endocr Pract. (2015) 21:725–33. 10.4158/EP14437.OR 25786553

[84] Yefet E Twafra S Shwartz N Hissin N Hasanein J Colodner R. Inverse association between 1,5-anhydroglucitol and neonatal diabetic complications. Endocrine. (2019) 66:210–9. 10.1007/s12020-019-02058-w 31435861

[85] Nelson RK Poroyko V Morowitz MJ Liu D Alverdy JC. Effect of dietary monosaccharides on *Pseudomonas aeruginosa* virulence. Surg Infect. (2013) 14:35–42. 10.1089/sur.2011.063 23451729 PMC3601807

[86] Lesman-Movshovich E Lerrer B Gilboa-Garber N. Blocking of *Pseudomonas aeruginosa* lectins by human milk glycans. Can J Microbiol. (2003) 49:230–5. 10.1139/w03-027 12795411

[87] Pelley JW Glycolysis and Pyruvate Oxidation. St. Luis: Elsevier's Integrated Biochemistry (2007). p. 47–53.

[88] Arthur PG Kent JC Hartmann PE. Metabolites of lactose synthesis in milk from women during established lactation. J Pediatr Gastroenterol Nutr. (1991) 13:260–6. 10.1097/00005176-199110000-00004 1791501

[89] Alderete TL Autran C Brekke BE Knight R Bode L Goran MI. Associations between human milk oligosaccharides and infant body composition in the first 6mo of life. Am J Clin Nutr. (2015) 102:1381–8. 10.3945/ajcn.115.115451 26511224 PMC6546222

[90] Charbonneau MR O'Donnell D Blanton LV Totten SM Davis JC Barratt MJ. Sialylated milk oligosaccharides promote microbiota-dependent growth in models of infant undernutrition. Cell. (2016) 164:859–71. 10.1016/j.cell.2016.01.024 26898329 PMC4793393

[91] Bhargava P Li C Stanya KJ Jacobi D Dai L Liu S. Immunomodulatory glycan LNFPIII alleviates hepatosteatosis and insulin resistance through direct and indirect control of metabolic pathways. Nat Med. (2012) 18:1665–72. 10.1038/nm.2962 23104131 PMC3493877

[92] Lagström H Rautava S Ollila H Kaljonen A Turta O Mäkelä J. Associations between human milk oligosaccharides and growth in infancy and early childhood. Am J Clin Nutr. (2020) 111:769–78. 10.1093/ajcn/nqaa010 32068776 PMC7138667

[93] Sprenger N Lee LY De Castro CA Steenhout P Thakkar SK. Longitudinal change of selected human milk oligosaccharides and association to infants' growth, an observatory, single center, longitudinal cohort study. PLoS ONE. (2017) 12:e0171814. 10.1371/journal.pone.0171814 28182762 PMC5300226

[94] Larsson MW Lind MV Laursen RP Yonemitsu C Larnkjær A Mølgaard C Human milk oligosaccharide composition is associated with excessive weight gain during exclusive breastfeeding-an explorative study. Front Pediatr. (2019) 7:297 10.3389/fped.2019.00297 31380329 PMC6657391

[95] Puccio G Alliet P Cajozzo C Janssens E Corsello G Sprenger N. Effects of infant formula with human milk oligosaccharides on growth and morbidity: a randomized multicenter trial. J Pediatr Gastroenterol Nutr. (2017) 64:624–31. 10.1097/MPG.0000000000001520 28107288 PMC5378003

[96] Marriage BJ Buck RH Goehring KC Oliver JS Williams JA. Infants fed a lower calorie formula with 2'FL show growth and 2'FL uptake like breast-fed infants. J Pediatr Gastroenterol Nutr. (2015) 61:649–58. 10.1097/MPG.0000000000000889 26154029 PMC4645963

[97] Rogero MM Calder PC. Obesity, inflammation, Toll-like receptor 4 and fatty acids. Nutrients. (2018) 10:432. 10.3390/nu10040432 29601492 PMC5946217

[98] Liu X Kris-Etherton PM West SG Lamarche B Jenkins DJ Fleming JA. Effects of canola and high-oleic-acid canola oils on abdominal fat mass in individuals with central obesity. Obesity. (2016) 24:2261–8. 10.1002/oby.21584 27804268 PMC5119743

[99] Yore MM Syed I Moraes-Vieira PM Zhang T Herman MA Homan EA. Discovery of a class of endogenous mammalian lipids with anti-diabetic and anti-inflammatory effects. Cell. (2014) 159:318–32. 10.1016/j.cell.2014.09.035 25303528 PMC4260972

[100] Silveira MB Carraro R Monereo S Tébar J. Conjugated linoleic acid (CLA) and obesity. Public Health Nutr. (2007) 10 (10A):1181–6. 10.1017/S1368980007000687 17903328

[101] Namazi N Irandoost P Larijani B Azadbakht L. The effects of supplementation with conjugated linoleic acid on anthropometric indices and body composition in overweight and obese subjects: a systematic review and meta-analysis. Crit Rev Food Sci Nutr. (2019) 59:2720–33. 10.1080/10408398.2018.1466107 29672124

[102] Viladomiu M Hontecillas R Bassaganya-Riera J. Modulation of inflammation and immunity by dietary conjugated linoleic acid. Eur J Pharmacol. (2016) 785:87–95. 10.1016/j.ejphar.2015.03.095 25987426

[103] Gaudet L Ferraro ZM Wen SW Walker M. Maternal obesity and occurrence of fetal macrosomia: a systematic review and meta-analysis. Biomed Res Int. (2014) 2014:640291. 10.1155/2014/640291 25544943 PMC4273542

[104] Voerman E Santos S PatroGolab B Amiano P Ballester F Barros H. Maternal body mass index, gestational weight gain, and the risk of overweight and obesity across childhood: an individual participant data meta-analysis. PLoS Med. (2019) 16:e1002744. 10.1371/journal.pmed.1002744 30742624 PMC6370184

[105] Weng SF Redsell SA Swift JA Yang M Glazebrook CP. Systematic review and meta-analyses of risk factors for childhood overweight identifiable during infancy. Arch Dis Child. (2012) 97:1019–26. 10.1136/archdischild-2012-302263 23109090 PMC3512440

[106] Centers for Disease Control Prevention Causes Consequences of Childhood Obesity. (2015). Available online at: https://www.cdc.gov/obesity/childhood/causes.html (accessed April 16, 2020).

[107] Kong L Nilsson IAK Brismar K Gissler M Lavebratt C. Associations of different types of maternal diabetes and body mass index with offspring psychiatric disorders. JAMA Netw Open. (2020) 3:e1920787. 10.1001/jamanetworkopen.2019.20787 32031649 PMC12578498

[108] Maayan L Hoogendoorn C Sweat V Convit A. Disinhibited eating in obese adolescents is associated with orbitofrontal volume reductions and executive dysfunction. Obesity. (2011) 19:1382–7. 10.1038/oby.2011.15 21350433 PMC3124611

[109] Laurent JS Watts R Adise S Allgaier N Chaarani B Garavan H. Associations among body mass index, cortical thickness, and executive function in children. JAMA Pediatr. (2019) 174:170–7. 10.1001/jamapediatrics.2019.4708 31816020 PMC6902097

[110] Amaral Y Marano D Oliveira E Moreira ME. Impact of pre-pregnancy excessive body weight on the composition of polyunsaturated fatty acids in breast milk: a systematic review. Int J Food Sci Nutr. (2020) 71:186–92. 10.1080/09637486.2019.1646713 31423865

